# Squamous cell papilloma a rare urinary bladder tumor, case report and operative video

**DOI:** 10.1016/j.eucr.2022.102074

**Published:** 2022-04-04

**Authors:** Ahmed Mohamed, Ibrahim A. Khalil, Maya Aldeeb, Bara Wazwaz, Ammar Al-Ani, Khalid Al-Jalham

**Affiliations:** a Department of Urology, Hamad Medical Corporation, Doha, Qatar; b Department of Medical Education, Family Medicine Residency Program, Hamad Medical Corporation, Doha, Qatar; c Department of Medical Education, Anatomical Pathology Residency Program, Hamad Medical Corporation, Doha, Qatar

**Keywords:** Squamous cell papilloma, Hematuria, Bladder tumors

## Abstract

Bladder noninvasive squamous lesions are usually rare; here, we are presenting a case of 39 years old male patient with a benign squamous cell papilloma. The tumor grossly presented as cauliflower mass mimicking squamous cell carcinoma, while histologically, the tumor showed extensive keratinization at its surface and showed no nuclear atypia or stromal invasion. This tumor is benign and extremely rare.

In this manuscript, we summarized a case of Squamous cell papilloma of the bladder with the first operative video for the transurethral resection of squamous cell papilloma with percutaneous management of associated urinary bladder stones.

## Abbreviations

TURB: (transurethral resection of the bladder)
CT: (Computed Tomography)
DRE: (digital rectal examination)
HPV: (human papilloma virus)

## 1 Introduction

Urinary bladder papillary lesions can be either neoplastic (benign or malignant) or non-neoplastic (Inflammatory, metaplastic changes). The urinary bladder is lined by urothelium, making neoplastic lesions mostly urothelial in origin. Malignant urothelial tumors are more common compared to benign tumors.^1^ Benign urothelial tumors include urothelial papillomas or inverted papillomas, while malignant tumors are urothelial carcinomas with or without invasion of the bladder wall.^1^^,^^2^

Squamous lesions of the bladder occurs significantly less to often, and Squamous cell carcinoma of the bladder represent only 2–5% of bladder tumors.^3^ Benign noninvasive squamous lesions of the bladder like squamous cell papilloma are extremely with few cases reported in the literature.^4^ This tumor endoscopically gave the picture of fungating mass and microscopically characterized by a papillary core with squamous epithelium covering without any cellular atypia or dysplasia.^5^ Here we present a case of squamous cell papilloma of the bladder with the first operative video for the transurethral resection of squamous cell papilloma with percutaneous management of associated urinary bladder stones.

## 2 Case presentation

**Fig. 1:**
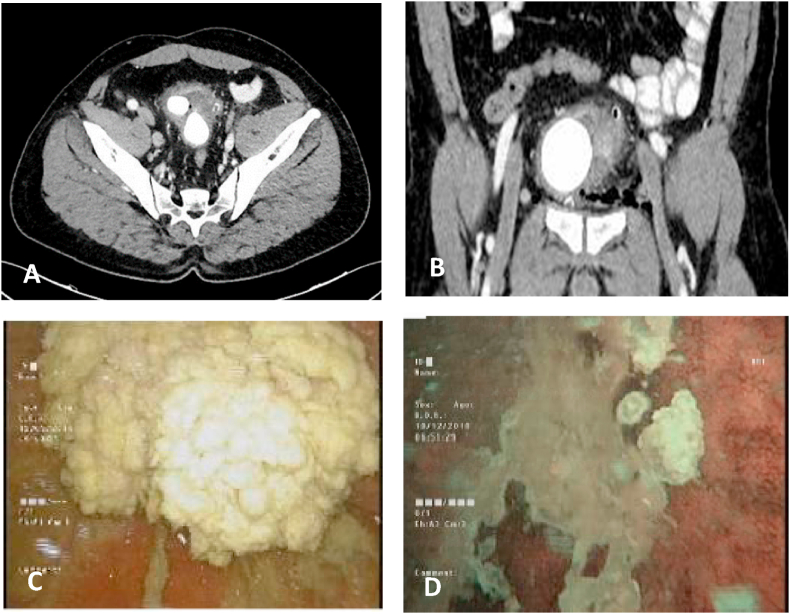
peri-operative CT scan and cystoscopy, A and B Ct scan of urinary bladder stone with a mass arising from anterior urinary bladder wall (axial and coronal views), C: first diagnostic cystoscopy showing extensive whitish exophytic cauliflower lesion, D:follow up cystoscopy showing whitish exophytic cauliflower lesion at the side of the primary tumor.

A 39-year-old healthy gentleman presented with macroscopic hematuria, associated with suprapubic abdominal pain, along with two years history of storage and voiding lower urinary tract symptoms. The patient underwent cystolithotomy for bladder stone three years prior to his presentation. He is a lifelong nonsmoker and has no family history of urinary tract malignancy. Physical examination is unremarkable. DRE showed average size prostate with no masses or nodules. Basic labs within normal limits. We proceeded with CT abdomen and pelvis with contrast as part of hematuria evaluation that showed irregular polypoidal thickening along with the anterior and lateral aspects of the urinary bladder wall, measuring up to 13 mm in thickness with three large intravesical stones 3 cm each (Fig. 1 (A-B) and 1B)). Diagnostic cystoscopy showed cauliflower extensive, whitish, and exophytic lesion found rising from the bladder's anterior wall and three sizeable urinary bladder stones (figure (1C)).

Our case surgical management was composed of three operations starting with *Trans*-urethral resection of the tumor (TURBT) to get tissue diagnosis; pathology result was squamous cell papilloma of the bladder. Then we attempted percutaneous cystolithotomy for the bladder stones after confirmation of no mass lesion recurrence by preoperative flexible cystoscopy one month after the first TURBT. The third endoscopic operative was three months later as follow up cystoscopy recurrence of whitish, exophytic cauliflower lesion at the primary site (figure (1D)) for which patient underwent the second TURBT again pathology confirmed the same finding of squamous cell papilloma, the operative Video contains the endoscopic surgical interventions (Video 1).

Supplementary video related to this article can be found at https://doi.org/10.1016/j.eucr.2022.102074

The following is/are the supplementary data related to this article:

Video 1 operative video of the transurethral resection of squamous cell papilloma of urinary bladder followed by percutaneous lithotripsy of associated bladder stones. .1 Video 1

## 3 Histopathology

**Fig. 2:**
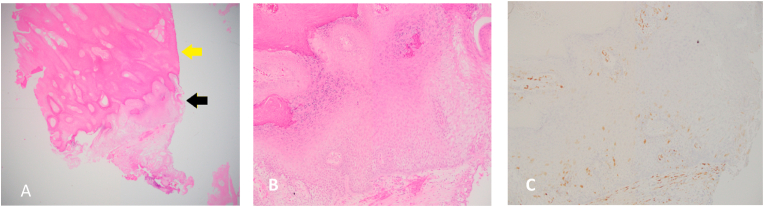
A: The lesion had a thick keratin layer (yellow arrow) overlying a stratified squamous epithelium (black arrow) (H&E stain magnification 20X). B: A closer look at the bland epithelium (magnification 100X).C: Few epithelial cells were positive (brown color) for P16 by immunohistochemistry. (For interpretation of the references to color in this figure legend, the reader is referred to the Web version of this article.)

Grossly the biopsy consisted of multiple tan, white, irregular, partially cauterized soft tissue fragments ranging from 0.4 to 1.2 cm in greatest dimension. Microscopically, the lesion had a thick keratin layer with underlying bland stratified squamous epithelium (Fig. (2A) and B)). There was no evidence of atypia or malignancy. By immunohistochemistry, the squamous epithelium was focally positive for P16 stain (Figure (2C)) and negative for HPV strain.

## 4 Discussion

Most urinary bladder tumors are urothelial neoplasms, while squamous cell lesions are rare to happen and can be either benign or malignant.^1^ Malignant squamous lesion includes squamous cell carcinoma in situ and invasive squamous cell carcinoma.^2^ Benign lesions include keratinizing squamous metaplasia, verrucous squamous hyperplasia, squamous cell papilloma, and condyloma acuminatum.^4^ Endoscopic visualization of these tumors will nearly be the same and resemble urothelial tumors; transurethral resection and histological analysis are needed to identify the cell of origin^3^. In some doubtful cases, as in our case, immunohistochemistry can differentiate squamous from urothelial tumors. Squamous cells express p63 and high molecular weight keratins.^5^

The importance of differentiating Squamous cell papilloma from other urinary bladder tumors arises from the benign nature of this tumor and that no treatment is needed compared to malignant urothelial and squamous tumors of the urinary bladder where aggressive treatment and resection are needed a possible role of local or systematic chemo or immunotherapy.

Here we present a rare case of recurrent Squamous cell papilloma of the urinary bladder confirmed by the microscopic and immunohistochemical diagnosis that allowed us to treat the stone percutaneously without risk of upgrading tumor a urinary bladder tumor due to begin nature with the operative video for the transurethral resection of the tumor and percutaneous management of associated urinary bladder stones.

## 5 Conclusion

Squamous cell papilloma is a rare benign urinary bladder tumor that cannot be differentiated endoscopically from malignant tumors but has characteristic microscopic and immunohistochemistry features. The diagnosis of this benign tumor prevents unnecessary aggressive treatment and allows percutaneous procedures of the urinary bladder.

## Declaration of competing interest

The authors declare that they have no financial or non-financial conflicts of interest related to the subject matter or materials discussed in the manuscript.
